# Methodological gaps in cervical LVA for Alzheimer’s disease: implications from regulatory intervention

**DOI:** 10.1097/JS9.0000000000003355

**Published:** 2025-09-09

**Authors:** Shichen Hu

**Affiliations:** Sichuan Taikang Hospital, Cardiovascular Center, Vascular Surgery, Chengdu, Sichuan, P.R. China

**HIGHLIGHTS**

- Regulatory interventions signal evidence deficits requiring methodological reforms.
- Surgical trials for neurodegenerative diseases must implement sham controls and long-term functional endpoints.
- Prospective complication registries are non-negotiable for vulnerable populations.

*Dear Editor,*

The pioneering study by Chen et al[1] on deep cervical lymphovenous anastomosis (LVA) for Alzheimer’s disease (AD) warrants rigorous methodological scrutiny, particularly in light of China’s recent regulatory prohibition[2]. Adhering to TITAN Guidelines for transparent AI use in scientific reporting[3], this correspondence critically examines three limitations that undermine clinical translation: Despite reporting MMSE improvement (median Δ = 2; *P* = 0.022), three fundamental flaws persist.

1. Lack of Controlled Design

In the absence of a control arm (N = 26), the observed cognitive gain cannot be dissociated from the placebo response inherent to invasive procedures. A recent sham-controlled trial in AD demonstrated that placebo-treated patients exhibited a mean 4.2-point MMSE improvement (95% CI 2.6-5.8) 1 month after surgery[4]. In the absence of comparable data for cervical lymphovenous anastomosis, future AD surgical trials must incorporate sham controls to isolate true therapeutic effects.

2. Inadequate Endpoint Selection

Short-term Mini-mental State Examination (MMSE) changes lack clinical relevance. The observed median postoperative MMSE = 5 (severe impairment) falls below the dementia threshold (MMSE<10)[5]. AD trials require 18-month CDR-SB or ADAS-Cog assessments[6].

3 Safety Oversights

Transient accessory nerve injury (7.7%) was underreported:

No electrophysiological confirmation[7].

No 6-month functional outcomes[8].

Novel Contributions to Literature

This critique provides:

First policy-integrated analysis linking regulatory intervention to specific evidence gaps.

Actionable solutions (Table 1) translating policy mandates into research standards.

**Table 1:** Evidence-to-policy alignment for future trials

Evidence Gap	Regulatory Requirement	Scientific Solution
Uncontrolled design	Sham-controlled trials	1:1 randomization (LVA vs. sham)
Short-term endpoints	18-month cognitive tracking	CDR-SB primary endpoint
Incomplete safety data	Independent monitoring	NSQIP-style registry

Ethical framework for surgical innovation in cognitively impaired populations.

## Data Availability

Any datasets are publicly available.

## Research registration unique identifying number (UIN)

Not involved.
